# Identifying Rewards Over Difficulties Buffers the Impact of Time in COVID-19 Lockdown for Parents in Australia

**DOI:** 10.3389/fpsyg.2020.606507

**Published:** 2020-12-17

**Authors:** Jane S. Herbert, Annaleise Mitchell, Stuart J. Brentnall, Amy L. Bird

**Affiliations:** ^1^Wollongong Infant Learning Lab, School of Psychology and Early Start, University of Wollongong, Wollongong, NSW, Australia; ^2^Molecular Horizons, University of Wollongong, Wollongong, NSW, Australia; ^3^School of Psychology, University of Waikato, Hamilton, New Zealand

**Keywords:** COVID-19, isolation, families, parenting, stress, resilience

## Abstract

**Objective:**

Physical isolation measures, known as lockdown or shelter-in-place, experienced during coronavirus disease 2019 (COVID-19) have the potential to cause psychological distress. This study was conducted to examine parents’ perceived stress and whether reports of rewards and challenges during lockdown impact stress.

**Methods:**

Data were collected using a cross-sectional online survey in New South Wales, Australia, across the 4-week lockdown. The survey was completed by 158 parents of children aged under 6 years. Stress was measured using the short form of the Perceived Stress Scale (PSS-4). Rewards and challenges were reported in response to two open-ended questions.

**Results:**

There was a weak negative correlation between PSS-4 scores and days in isolation (*r* = −0.167, *p* = 0.022), with parents who had spent longer in isolation reporting fewer stress symptoms. The relationship between time in isolation and stress was moderated by the degree to which parents described more rewards than challenges: parents who perceived high rewards and low challenges reported lower PSS-4 scores with more days in lockdown, whereas parents who perceived low rewards and high challenges reported higher PSS-4 scores with more days in lockdown. The moderation model examining associations between time in isolation and rewards ratio explained 13% of the variance in PSS-4 scores.

**Conclusion:**

Lockdowns are not uniformly or consistently negative experiences for parents. Identifying positive aspects of the experience may serve to buffer negative mental health risks across time. Understanding resilience strategies is critical for supporting current psychological wellbeing and to adequately prepare for future pandemic experiences.

## Introduction

As coronavirus disease 2019 (COVID-19) spread around the world, government containment measures changed the daily lives of millions of individuals. Workplaces, educational settings, and public activities were closed or limited, and physical distancing measures were implemented. While “lockdowns” or “shelter in place” directives were effective for restricting virus spread, concerns were increasingly voiced over the economic, social, and broader health impacts of these measures. Although COVID-19 is a global adverse event, the strength and speed of lockdown implementation has varied across countries. Personal experiences of lockdowns will also have varied widely depending on individual and family circumstances (Fegert et al., 2020). Understanding the risk factors for psychological wellbeing during this adverse event, and the factors that contribute to resilience, in which individuals successfully adapt or change in response to adversity (Dowrick et al., 2008), is of key importance for researchers and policy-makers alike.

COVID-19 has the potential to be a “perfect storm” for stress: exposure to uncertain multi-systemic health and economic stressors, in combination with prolonged isolation from usual social supports and coping mechanisms (Brooks et al., 2020; Douglas et al., 2020). Drawing on studies from previous pandemics such as severe acute respiratory syndrome (SARS) in 2013, Ebola in 2014, and H1N1 influenza in 2009 and 2010, a rapid review by Brooks et al. (2020) identified key stressors to include fears of infection, frustrations and boredom when movement is restricted, and distress resulting from inadequate access to supplies or information and financial losses. Even when not in directly infected zones, psychological distress is elevated during a disease epidemic, such as experienced by horse owners across Australian states during the equine influenza (Taylor et al., 2008). The effects of these stressors may be further magnified if typical strategies for managing adversity are disrupted. For example, the vast majority of individuals surveyed in an Australian population health study spontaneously identified their immediate family and parents (52%), or friends and neighbors (21%) when asked “How do you get through tough times?” (Taylor et al., 2010). With lockdowns disrupting the ways in which these supports can be accessed (e.g., the absence of face-to-face interactions), individuals may become more vulnerable to psychological stressors.

There is some evidence to suggest that having children is associated with higher levels of psychological distress for adults during health pandemics, although the duration of the isolation may be an important consideration in these findings. During the quarantines and movement restrictions of the equine influenza pandemic, which was eradiated in Australia over a period of about 4 months, adults with a child reported higher levels of psychological distress, as determined by the Kessler 10 Psychological Distress Scale, than adults with no children (Taylor et al., 2008). In contrast, no associations were observed between having children and reports of post-traumatic stress disorder symptoms or depression after the short duration (median 10-day) quarantine experienced as the result of SARS outbreaks (Hawryluck et al., 2004).

The experience of prolonged isolation during COVID-19 is unprecedented, and may create unique stressors for parents and families. These stressors include attempting to mitigate lost opportunities for children’s learning and social interactions, reduced daily structure, reduced physical activity, and increased screen time use (Fegert et al., 2020; Gassman-Pines et al., 2020; Guan et al., 2020; Wang et al., 2020). There is early evidence to suggest that mothers of young children are experiencing higher rates of depression and anxiety through COVID-19 (between 30 and 40% of mothers reporting symptoms in the clinical ranges). Previous experience of mental health difficulties, marital discord, and financial strain were significant predictors (Cameron et al., 2020). We know very little, however, about the process by which parents might be coping with COVID-19 stress, and how this might exacerbate or buffer against the experience of these stressors.

Understanding families’ experiences during COVID-19 is also important for understanding the impact on parenting and children’s mental health (Fegert et al., 2020). Initial reports from the COVID-19 pandemic indicate there have been significant increases in school-aged children’s depression symptoms during lockdown in the UK, relative to 18 months earlier (Bignardi et al., 2020). Initial reports from Japan further suggest significant increases have occurred in parenting stress during COVID-19 school closures (Hiraoka and Tomoda, 2020). Any changes in psychological wellbeing of parents and children over lockdown are likely to impact parenting behavior. The pre-COVID-19 scientific literature highlights the negative impact of parent stress on parenting behaviors and long-term child outcomes (Barroso et al., 2018).

How might parents be coping with the adversity of prolonged isolation during lockdowns and social isolation? The extensive literature on psychosocial resilience to adversity highlights several key mechanisms: (1) the experience of positive and negative emotions; (2) cognitive flexibility or positive appraisal; (3) finding meaning; (4) connecting with social supports; (5) engaging in active coping (e.g., exercise) (Southwick et al., 2005). These include both specific coping tools (social support and exercise), as well as cognitive coping processes (flexibility and reappraisal). In the COVID-19 context, Coyne et al. (2020) suggest that psychological flexibility will be demonstrated by a parent’s capacity to identify rewards or positives in the “small things” (e.g., incremental exercise), to find meaning in these moments, and to connect with others. Potential beneficial consequences of the lockdown include opportunities for stress-related growth and development by individuals and in family relationships (Fegert et al., 2020). In support, Daks et al. (2020) found that greater parent psychological flexibility predicts higher family cohesion and lower COVID-19–related stress.

Although this has not yet been examined empirically in the COVID-19 context, there is some evidence that models of positive resilience and psychological flexibility apply to large-scale population-level crises. For example, research conducted by Fredrickson et al. (2003) following the September 11, 2001 terrorist attacks in the USA revealed that positive emotions such as gratitude, interest, and love were crucial elements that helped resilient individuals to thrive despite adversity. Notably, these individuals were not exclusively reporting positive emotions, but were demonstrating the capacity to acknowledge both negative emotions—including anger, fear, and disgust—with more positive experiences. In contrast, early COVID-19 data from Italy indicates that parents who rated restrictions due to lockdown (e.g., limited activities, finding time to spend with partner) as more difficult also experienced higher stress (Spinelli et al., 2020). Data from the US have also found that parents’ report of support and perceived control were associated with reduced perceived stress (Brown et al., 2020). Interestingly, Brown et al. also asked parents a single open-ended question about the impacts of COVID-19. Most parents described a range of stressors, with approximately 10% of parents reporting a positive change of spending more time with family; however, these ratings were only presented descriptively and were not examined in relation to parents’ stress. Specifically prompting parents for both challenges and rewards may help to elucidate additional positive mechanisms, and allow for quantitative analysis. Given the likelihood of ongoing lockdowns and physical isolation measures in many countries, understanding family resilience processes during COVID-19 is critical.

While COVID-19 has had an international impact, the arrival date of the virus in each country and the initial responses from each government has varied widely. COVID-19 arrived in Australia well after it had become established in China and across Europe, with the first confirmed case reported on January 25, 2020. The Australian Federal government announced mandatory closure of “non-essential” services on March 23, with a further directive on March 31 requiring people to stay at home unless they had a “reasonable excuse.” Restrictions began to loosen slightly from May 1, 2020 with the advice changing to allow two unrelated adults and their children to visit another’s family home. However, other restrictions (e.g., working from home, returning to school, bans on large gatherings) remained in place, and continue even months later to some degree. Compared with other countries (e.g., Italy, New Zealand), restrictions were not strictly enforced, creating some variability in time of entry and degree of exposure to lockdown. This represents a unique context in which to examine the impact of lockdown on stress in families.

The aim of the current study is to examine parents’ perceived stress and their descriptions of the challenges and rewards experienced during the COVID-19 lockdown in Australia. We focused on the parents of children under the age of 6 years due to the wide variety of educational disruptions that may have occurred for children who were in formal education at the time of lockdown. Moreover, the preschool years are widely recognized as a sensitive period for long-term developmental outcomes (Caspi et al., 2016). The aims of the current study were to (1) provide an important window into the experience of families during this completely unprecedented and novel global event by asking about rewards and challenges, and (2) allow examination of whether specific types of rewards and challenges impact on parenting stress. Existing models and research highlights that resiliency is associated with psychological flexibility and a capacity to perceive meaning in adversity. In the COVID-19 context, this is proposed to be reflected in a parent’s capacity to find meaning in daily moments, connect socially, and a capacity to recognize positive alongside negative aspects of the pandemic (Coyne et al., 2020). In addition, the degree to which a parent considers their child’s needs and perspective through adversity is well recognized as a positive parenting construct, including through the COVID-19 pandemic (Bate and Malberg, 2020). Existing research has identified an association between cumulative COVID-19 hardship (job loss, income loss, caregiver burden, and household illness) and both parent and child mental health (Gassman-Pines et al., 2020). The less globally restrictive approach to lockdown in Australia allows us to also consider cumulative time spent in lockdown as a COVID-19 stressor, with parents’ self-reported perceived stress the mental health outcome. The current study therefore examines whether the impact of COVID-19 stressors (time in lockdown, job loss, caregiver burden) on parents’ perceived stress is buffered or moderated by the degree to which parents perceive positive aspects of lockdown, connect socially during lockdown, and consider the rewards and challenges from their child’s perspective. An alternative explanation to buffering may be a mediation pathway whereby COVID-19 stressors predict changes in the degree to which parents perceive positive aspects of lockdown or connect socially, which in turn predicts parents’ perceived stress. Both models will be tested, with a lack of extant research preventing *a priori* hypotheses.

## Materials and Methods

### Study Design

To understand the effects of COVID-19 lockdown on parents, we prepared a web-based cross-sectional survey to be distributed to a convenience sample of parents of children aged under 6 years in the state of New South Wales, Australia. Participants were asked to report their demographic data, the date they chose to enter social isolation, household composition and employment status before and during the pandemic, and a standardized measure of their perceived stress level. Participants were also asked two open-ended questions about the challenging and rewarding aspects of the COVID-19 situation.

The survey was distributed via direct email and social media advertising in two waves. During the first wave (from April 2), a survey invitation was sent to previous participants or those interested in infant research at the Wollongong Infant Learning Lab. This invitation was sent to 131 families, who were also encouraged to share the survey link. During a second wave of data collection (from April 26), the invitation was extended to members and followers of the large local museum for children aged birth to 10 years, Early Start Discovery Space. Both the infant lab and the children’s museum are located in the same building at the University of Wollongong. Although families were only able to participate in the survey once, some families will have received an invitation to participate during both waves. The survey closed on April 30, in line with the first easing of the social isolation restrictions.

### Participants

A total of 200 individuals indicated their consent to participate, but 42 of these did not complete the initial demographic questions. The remaining 158 participants completed the survey: *n* = 38 during the first recruitment wave; *n* = 120 completed the survey during the second recruitment wave. Of the 158 respondents, 143 provided full data for the current study. Specifically, 15 individuals from the original sample had not included a date for when they began isolation (or stated that they were not practicing this), and one of these individuals had also not completed the PSS-4 or given a response on the rewards and challenges open-ended questions. Preliminary analyses indicated that there was no difference in sociodemographic variables between individuals who responded to all questions and those who did not.

Survey respondents were predominately female (97%), and married or in a *de facto* relationship (93%). The majority of respondents reported their current housing arrangement as paying off a mortgage (59%) or paying rent (26%), with the remainder living rent free or paying off family. Respondents were primarily employed and working full time (16%), part time (40%), or on paid maternity leave (19%) before COVID-19. The remainder were not in the workforce (16%) or unemployed and looking for work (9%).

### Measures

#### Demographic Information and COVID-19 Hardships

Demographic variables included gender, date of birth, postcode, and marital status. Household questions asked about housing arrangement (e.g., renting, ownership, living rent free), and the number and age of people living in the house. Employment questions included current employment status (employed: full time, part time, away from work; unemployed: looking for full-time work, looking for part-time work; not in the work force), occupation, and whether anyone in the household experienced a COVID-19–related change in employment status (yes or no). COVID-19 questions included whether social isolation was currently being practiced, and if so, when their isolation began. This question was scored as the number of days between entering into isolation and completing the survey, with a score of 0 being given if social isolation had not occurred. COVID-19 caregiver burden was conceptualized as the number of children aged under 6 years living in the household.

#### Perceived Stress Scale

Stress was reported using the 4-item version of the Perceived Stress Scale (PSS-4), which uses general questions about psychological stress rather than focusing on a specific experience (Cohen et al., 1983). Although originally a 14-item scale, the PSS-4 has been identified as a useful tool when data are being conducted quickly and remotely (Herrero and Meneses, 2006; Mañanes et al., 2016), and has been normed internationally (Warttig et al., 2013; Vallejo et al., 2018). The scale asks the participant to report on their thoughts and feelings of control over life events and confidence in dealing with these experiences during the last month. Responses are given on a scale from 0 (never) to 4 (very often). PSS-4 scores are obtained by summing together the scores of the four questions, with the middle two items being reverse scored. The maximum score on this measure is 16. This measure has a reliability level of α = 0.82 (Mitchell et al., 2008). The reliability in the current sample (α = 0.78) was comparable.

#### Rewards and Challenges

Given the established value of asking about positive and negative experiences when evaluating wellbeing (e.g., van der Zwan et al., 2017), and the use of open-ended questions for understanding how individuals manage adversity (Taylor et al., 2010), the following questions were included in the survey: “*Have there been challenging parts? If so, what have been the most challenging parts?*” and “*Have there been rewarding parts? If so, what have been the most rewarding parts?*” These questions have previously been used in a longitudinal study of 113 women to capture the range of positive and negative experiences during pregnancy (McNamara et al., under review). To direct respondents to focus on their pandemic experience in the current study, these questions were preceded by the statement “The current COVID-19 situation has caused many changes for individuals and families within our community.” Respondents were free to write as much or as little as they wanted for these questions.

### Procedure

The questionnaire was hosted on Qualtrics. No questions were compulsory to answer, and the questionnaire was voluntary and non-commercial. There was no individual incentive for participation. A donation of $100 was given to a local housing support charity in recognition of the time families had given in completing the voluntary survey.

The study was approved by the University of Wollongong Human Research Ethics Committee (2018/399). Electronic informed consent was obtained from each participant before they started the survey. Participants could withdraw from the survey at any time by closing their browser and only surveys that were fully completed were included in the analysis.

### Coding of Rewards and Challenges

Responses to the open-ended questions were coded using a scheme developed from key parenting themes outlined in Bradley (2007). These themes encompass a range of ways that parents support children during challenging circumstances that threaten wellbeing and subsequent development (including war, natural disasters, loss, and abuse). These themes were derived from models describing the core tasks of parenting, and from the empirical literature examining parenting and child development through challenging circumstances (Bradley, 2007). The themes, their descriptions, and examples from the rewards and challenges questions in the current sample are described in Table 1. In addition to these themes, each code was further categorized as referring to either the child (e.g., “my child has been experiencing a lot of anxiety”) or the parent (e.g., “I have been really anxious over this whole lockdown”). The data were initially divided into idea units and then coded by one researcher (A.B.). A second researcher (A.M.) coded 20% of the sample for inter-rater reliability. Inter-rater reliability was calculated using Cohen’s Kappa, with a mean estimate of 0.79, considered substantial agreement.

**TABLE 1:** Coding of parental rewards and challenges based on Bradley (2007) framework for parenting under challenging circumstances.

Theme	Description	Challenge example	Reward example
Safety	Provision of sustenance and basic necessities for health and life; including food, exposure to illness, protection from imminent harm	*“I’m an essential worker and it’s stressful thinking I could get sick and pass it on”*	*“It’s good to be in lockdown and safe from the virus”*
Socioemotional support	Provision of social and emotional support; including communication, encouragement, discipline, and warmth	*“My child has anxiety anyway and this has made things a lot worse”*	*“Having a lot of fun together, just enjoying being together”*
Stimulation (instruction)	Provision of stimulating experiences for the child; including recreational activities, toys, and more formal learning opportunities	*“My child can’t go to day-care”*	*“Being able to be involved in my child’s learning every day (e.g., toilet training)”*
Monitoring	Capacity to gather data about the child; including proximity or contact to enable this	*“I don’t even know what my kids are doing all day, I’m too busy working from home”*	*“Seeing my child reach milestones that I would have missed otherwise”*
Structure	An environment that provides structure, routine, and organization	*“Everything feels very chaotic in the household”*	*“Being able to slow down and spend more time together every day”*
Social connectedness	Connection with peers, extended family, and other social and community networks	*“Not being able to see my parents”*	*“Connecting with everyone over Zoom and Skype”*

#### Data Reduction for Rewards and Challenges

Due to a zero-inflated distribution, parents’ references to safety challenges and rewards were considered as dichotomous yes/no variables. Because of very low numbers of parental references, we also collapsed four of the rewards and challenges categories into two. Having proximity and contact with a child to observe them (the monitoring theme) is closely linked with structure, organization, and daily routine of a household (the structure theme); so these categories were combined. Similarly, providing and receiving socioemotional support is closely linked with wider socioemotional connections. For example, “my partner really helps me relax” would be coded as socioemotional support, whereas “it’s great being able to talk with my parents on Zoom” would be social connectedness. These two categories were also combined in subsequent analyses. In line with hypotheses, a variable was created for total child references as a proportion of total references for both challenges and rewards (child ratio). An additional ratio was calculated for the total number of reward descriptors divided by the total number of descriptors (rewards ratio).

### Data Analysis

Skewness and kurtosis values were calculated (alongside histogram inspections) for each of the six continuous descriptive variables: social/emotional, structural/monitoring, and stimulation for both challenges and rewards. Because of positive skewness, these descriptor variables were transformed using square root transformations (recommended for zero-inflated count distributions). PSS-4 scores were logit transformed. Transformed variables were used in subsequent correlational analyses and untransformed variables in regression analyses which are generally more robust to skewness. Pearson correlation coefficients were calculated to examine associations among coded descriptor variables and with PSS-4 scores and parental age (continuous). Chi square analyses were calculated to examine associations of recoded dichotomous descriptor variables (safety) with one another. Independent sample *t*-tests were calculated to examine potential differences in descriptor variables and PSS-4 scores across the two samples. One-way ANOVAs examined differences in PSS-4 scores based on changes in employment following COVID-19. The association of PSS-4 with COVID-19 hardships (days in isolation and caregiver burden) was performed using R (R Core Team, 2020) and plotted using ggplot2 (Wickham, 2016). All other analyses were conducted in SPSS (Version 26).

Mediation and moderation hypotheses were tested using a bootstrapping method (Hayes, 2009) with the Process macro for SPSS (Hayes, 2018). This is a contemporary approach for mediation and moderation modeling that allows non-normality and asymmetry, and balances power and validity concerns (Hayes, 2013; Hayes and Preacher, 2013). For the mediation model, the bias-corrected bootstrap confidence intervals for each of the indirect effects were based on 5,000 bootstrap samples, using 95% confidence intervals (Preacher and Hayes, 2004, 2008). The indirect pathway is supported when the confidence intervals do not cross zero.

## Results

### Descriptive Statistics

Means, *SD*s, and ranges for parental descriptors, days spent in isolation, and PSS-4 scores are shown in Table 2. Scores on the PSS-4 ranged from 1 to 14, with a mean of 6.31 (*SD* = 2.85). The duration spent in social isolation at the time of survey completion ranged from 5 to 71 days (*M* = 33 days, *SD* = 14 days).

**TABLE 2:** Means, *SD*s, and ranges for maternal descriptors and perceived stress.

	Mean (*SD*)	Range	*n* (%)
**Challenges**			
Safety	0.39 (0.82)	0–5	
Social/emotional	1.73 (1.78)	0–10	
Structural	0.96 (1.10)	0–6	
Stimulation/monitoring	1.20 (1.37)	0–9	
**Rewards**			
Safety	0.23 (0.52)	0–3	
Social/emotional	0.44 (0.79)	0–4	
Structural	1.32 (1.06)	0–5	
Stimulation/monitoring	0.82 (1.24)	0–7	
Child ratio	0.24 (0.24)	0–1	
Reward ratio	0.40 (0.17)	0–0.89	
PSS-4	6.31 (2.85)	0–14	
**COVID-19 stressors**			
Caregiver burden (number of children < 6 years living in household)	1.46 (0.65)	0–5	
Time in isolation (days)	33.71 (13.86)	5–71	
Change in employment			77 (45%)

### COVID-19 Hardship and Sociodemographic Predictors of Perceived Stress

**FIGURE 1:**
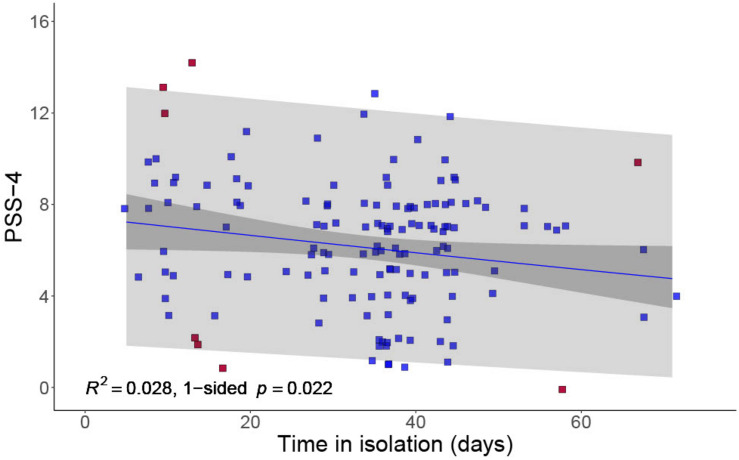
Negative association between PSS-4 and duration spent in COVID-19 isolation. As there were some instances where different individuals reported the same PSS-4 scores and duration in isolation, points on this figure have been jittered for clarity. 95% confidence and prediction bands are shown. The eight points highlighted in red were identified as potentially influential by viewing a histogram of Cook’s distance. With these points excluded, the *p*-value is 0.036.

Associations were first examined between PSS-4 scores, sociodemographic variables, and COVID-19–related variables. There were no significant associations between PSS-4 scores and maternal age, number of people living in the household, or number of preschool children in the household. There was no significant difference in PSS-4 scores between those who had experienced a change in employment (*M* = 6.68, *SD* = 3.07) and those who had not (*M* = 6.01, *SD* = 2.63), *t* = −1.46, *p* = 0.147. Parents who had experienced a change in employment reported fewer social emotional rewards related to COVID-19, *t* = 1.27, *p* = 0.004. A weak but significant negative correlation was found between days spent in isolation and PSS-4 scores, *r* = -0.167, *p* = 0.022 (one-sided *t*-test against null hypothesis of zero slope that there is no *increase* in stress over time), with parents having spent longer in lockdown reporting fewer symptoms of perceived stress (see Figure 1). Days in isolation was the only COVID-19 hardship variable significantly related at the univariable level with parents’ PSS-4 scores, and is therefore the only hardship variable considered in subsequent moderation and mediation analyses.

### Comparison Across Data Collection Waves

Independent-samples *t*-tests were also conducted to examine differences in means of continuous variables across the two waves of data collection. There was a significant difference in stimulation rewards *t* = -1.915, *p* = 0.035, social emotional rewards *t* = -1.992, *p* = 0.002, structural rewards *t* = -2.507, *p* < 0.0001, and the rewards ratio *t* = -2.439, *p* = 0.016. In all three instances, the cohort that was recruited from the infant research lab (wave 1 data) had lower means than the children’s museum cohort (wave 2 data). There was no difference in the number of children aged under 6 years living in the household, *t* = -0.374, *p* = 0.709. There was a significant difference in PSS-4 scores across the two waves, with parents from the first wave showing higher PSS-4 scores (*M* = 7.19, *SD* = 3.14) than those in the second wave (*M* = 6.04, *SD* = 2.71), *t* = 2.17, *p* = 0.03. There was a significant difference in parental age, with the parents in the first wave younger (*M* = 32.92, *SD* = 5.08) than those in the second wave (*M* = 35.85, *SD* = 5.22), *t* = -3.00, *p* = 0.003. Given these differences, participant wave was included as a covariate in subsequent regression analyses.

### Parental Descriptors of Challenges and Rewards and Perceived Stress

Pearson correlation coefficients were then calculated between the continuous descriptive variables and PSS-4 scores. Spearman rho correlation coefficients were calculated between parents’ references to safety as a challenge or reward, and PSS-4 scores (see Table 3). Results indicated that no significant associations between PSS-4 scores and any of the category-specific parental descriptors of rewards or challenges, with the exception of a marginally significant Spearman correlation coefficient between parental references to safety challenges and PSS-4 scores, *r* = 0.158, *p* = 0.05. Parents who reported higher perceived stress symptoms were more likely to describe safety as a challenge. A significant negative Pearson correlation coefficient was also found between the reward ratio and PSS-4 scores, *r* = −0.233, *p* = 0.003. Parents who described more a greater proportion of rewards than challenges reported lower symptoms of perceived stress.

**TABLE 3:** Associations of perceived stress with maternal descriptors of challenges and rewards: Pearson and Spearman rank-order correlations and χ^2^.

	Challenges				Rewards				Ratios		Stress
			
	Safety	Social/emotional	Structural	Stimulation	Safety	Social/emotional	Structural	Stimulation	Child ratio	Reward ratio	PSS-4
**Challenges**											
Safety	–	0.015	−0.017	−0.041	χ^2^ = 0.491	0.006	−0.004	−0.025	−0.25**	−0.204*	0.158*
SE		–	0.021	0.201*	0.091	0.235**	0.240**	0.232**	0.042	−0.223**	0.133
Structural			–	0.081	0.048	0.001	0.205*	0.045	−0.132	−0.246**	0.092
SM				–	−0.039	0.015	0.111	0.142	0.397****	−0.321****	−0.069
**Rewards**											
Safety					–	0.018	0.040	0.128	−0.117	0.246**	0.128
SE						–	−0.010	0.287****	−0.084	0.346****	−0.036
Structural							–	−0.109	−0.134	0.213**	−0.153
SM								–	0.109	0.411****	−0.022
Child ratio									–	−0.167*	−0.009
Reward ratio										–	−0.233**
PSS-4											–

*p < 0.05, **p < 0.01, ****p < 0.0001; SE, socioemotional support and social connectedness; SM, stimulation and monitoring.

### Regression Analyses: Testing Mediation and Moderation

The association between time in isolation, perceived stress, and rewards ratio was further explored. Potential mediation and moderation pathways were tested. There was a significant difference in reward ratio values across the two waves, so this was included as a covariate in all models.

#### Mediation Model

The independent variable was days in isolation, the dependent variable PSS-4 scores, and the mediator rewards ratio. The unstandardized coefficients, SEs, and 95% CIs are shown in Table 4. The indirect effect through the rewards ratio was not statistically different from zero, with 95% CIs crossing zero (*z* = −0.0005, 95% CI −0.0151 to 0.0128).

**TABLE 4:** Model coefficients for the simple mediation analysis of days of isolation on perceived stress, mediated through parental rewards ratio with sample as covariate.

	Consequent											

	*M* (rewards ratio)						*Y* (PSS-4)					
Antecedent	Coefficient	SE	*t*	*p*	LLCI	ULCI	Coefficient	SE	*t*	*p*	LLCI	ULCI
*X* (days of isolation)	0.0002	0.0017	0.0888	0.9294	0.1510	0.4029	−0.0066	0.0284	−0.2319	0.8170	−0.0628	0.0496
*M* (rewards ratio)	–	–	–	–	–	–	−3.4518	1.4085	−2.4508	0.0155	−6.2366	−0.6670
Covariate (wave)	0.0711	0.0560	1.2701	0.2062	−0.0396	0.1817	−0.8683	0.9380	−0.9257	0.3562	−2.7229	0.9863
Constant	0.2769	0.0637	4.3458	0.0000	0.1510	0.4029	9.4669	1.1314	8.3674	0.0000	7.2299	11.7039
	*R*^2^ = 0.0363 *F*(2, 140) = 2.6387, *p* = 0.0750						*R*^2^ = 0.0781 *F*(3, 139) = 3.9264, *p* = 0.01					

#### Moderation Model

The independent variable was days in isolation, the dependent variable PSS-4 scores, and the moderator variable rewards ratio. The model explained 13% of the variance in PSS-4 scores [*F*(3, 139) = 6.81, *p* = 0.0003, *R*^2^ = 0.128]. Unstandardized coefficients, SEs, and 95% CIs are shown in Table 5. Days spent in isolation was a significant predictor of PSS-4 scores [*B* = 0.092, *t*(139) = 2.12, *p* = 0.04]. Parental rewards ratio was a marginally significant predictor of PSS-4 scores [*B* = 7.31, *t*(139) = 1.87, *p* = 0.06]. The interaction effect was statistically significant and different from zero [*B* = −0.309, *t*(139) = −2.98, *p* = 0.003]. Therefore, the effect of days in isolation on perceived stress depends on the degree to which parents perceive rewards more than challenges (see Supplementary Figure 2).

**TABLE 5:** Model coefficients for testing moderation of the relationship between days of isolation and perceived stress by parental rewards ratio.

	Coefficient	SE	*t*	*p*	LLCI	ULCI
Days of isolation	–0.0031	0.0276	–0.1109	0.9119	–0.0576	0.0515
Rewards ratio	–2.8734	1.3769	–2.0868	0.0387	–5.5960	–0.1508
Covariate (wave)	–1.2998	0.9191	–1.4143	0.1595	–3.1171	0.5175
Days of isolation × rewards ratio	–0.3313	0.1046	–3.1688	0.0019	–0.5381	–0.1246
Constant	8.7212	1.6472	5.2944	0.0000	5.4641	11.9783
	*R*^2^ = 0.1407 *F*(4, 138) = 5.6467, *p* = 0.0003					

## Discussion

The experience of lockdown is not uniformly or consistently negative across time for all individuals. Our findings provide a more nuanced view of the existing literature showing greater cumulative COVID-19 hardships predict poorer parental mental health (Gassman-Pines et al., 2020). Within our sample of Australian parents of preschool children, the relationship between COVID-19 hardship (defined as days spent in isolation) and parents’ perceived stress varied as a function of the degree to which parents perceived rewards over challenges during lockdown. For parents who perceived greater rewards, longer time in isolation was associated with *lower* stress, whereas for parents who perceived greater challenges, longer isolation was associated with *higher* stress. Contrary to predictions, the specific degree to which parents highlighted social and emotional connection and support, or considered the rewards and challenges of lockdown from their child’s perspective, was not related. Essentially, our findings indicate that being able to identify more rewarding aspects compared with challenging aspects of the lockdown may serve to build parents’ resilience, buffering against the potential negative psychological impacts of longer in lockdown.

This cross-sectional survey data provides the first evidence that decreased psychological wellbeing is not an inevitable consequence of time in lockdown. Although a small body of research from the previous SARS pandemic suggests that longer quarantine times may be associated with poorer mental health outcomes (e.g., Hawryluck et al., 2004; Reynolds et al., 2008), it has been proposed that the negative effects could be mitigated by providing individuals with as much information as possible about the expected duration of isolation and virus spread, as well as ensuring adequate access to supplies (see Brooks et al., 2020 for review). At the time of our survey during the “first wave” of COVID-19, Australia had experienced relatively few cases of COVID-19 infections which resulted in a relatively short, 4-week, lockdown that was not strictly enforced. We speculate that a sense of individual control was restored for some parents in response to the generally well-communicated, contained, and apparently effective lockdown.

Our findings build from research showing that offsetting negative emotions with more positive emotions helps resilient individuals to thrive despite having experienced adversity (Folkman, 1997; Folkman and Moskowitz, 2000; Fredrickson et al., 2003). Being able to identify positive experiences during the major life disruption caused by COVID-19, not just after the event, appears to play an important role in supporting current psychological wellbeing. Further research is needed to understand the specific mechanisms by which some parents are able to perceive rewards and find meaning during adversity, but this may include constructs from positive psychology such as character strengths, values, grit, flow, and optimism (Seligman, 2011) and/or constructs such as psychological flexibility and self- and other-compassion (Coyne et al., 2020; Daks et al., 2020). Although no causality can be inferred from the current data, they do support many of the recent calls for clinicians to support families to develop psychological flexibility through mindfulness and self-compassion practice (Coyne et al., 2020).

Given the well-documented effects of stress on parenting (Barroso et al., 2018), we speculate that this positive attitude during shared adversity may enhance the longer-term growth and strength of the parent–child relationship. Prime et al. (2020) propose a model of risk and resilience for families through COVID-19. They emphasize the importance of measuring multiple aspects of the family system as potential sources of resilience, including sibling, parent-to-parent, and parent–child communication, beliefs, and organization. It remains to be seen how the rewards to challenges ratio identified here as a resilience factor for the individual primary caregiving parent is reflected in relationships across the family system and its impact on child functioning. Early data indicate that caregiver burden through COVID-19 impacts parent–child closeness and conflict (Russell et al., 2020), and that psychological inflexibility predicts more caustic parenting and greater child distress (Daks et al., 2020). This area warrants further investigation with longitudinal studies, given that a better understanding of this coping approach offers opportunities for early intervention. Further research is also needed to examine stressors and specific resilience pathways for families who may be at increased risk for mental health impacts through COVID-19, including families of children with special needs (Asbury et al., 2020) or with health problems (Fontanesi et al., 2020; Yuan et al., 2020), low-income families, and families where a parent is an essential worker (Coyne et al., 2020). In addition, COVID-19 presents a number of hardships that were not able to be measured within the current survey (e.g., sleep difficulties; Zreik et al., 2020) and should be explored in future research.

Despite being in lockdown, overall the parents in our study did not show elevated levels of perceived stress compared with the population norms reported by Warttig et al. (2013) from the England sample with a similar age profile of 30–44 years (*M* = 6.05, *SD* = 3.16, *N* = 596). Our findings are in contrast to early data from Canada showing that clinical rates of depression and anxiety are prevalent in 30–40% of expectant mothers and mothers of children aged 0–8 years (Cameron et al., 2020). It is important to recognize that the current study did not use clinical measures of mental health symptoms with established clinical cut-offs, but instead captured a generalized measure of perceived stress over the past month. For some of our families who completed the survey earlier in lockdown, the 1-month time period given as reference in the PSS-4 will have covered events before lockdown. While the weeks immediately before lockdown were potentially less stressful, the virus was already well established in Australia and around the world early in 2020. Regular news reporting and social media updates of virus spread were likely impacting on psychological wellbeing well in advance of lockdown implementation.

An alternative possibility for stress levels not being heightened overall in our sample, despite the pandemic, relates to the fixed order design of our questionnaire. Specifically, we asked respondents to identify any challenging parts and then any rewarding parts of the current COVID-19 situation, immediately before the PSS-4. This question ordering may have encouraged respondents to start engaging in more positive cognitive appraisals of their situation before evaluating their own wellbeing. Out of the entire sample, only three individuals were unable to identify any rewarding parts, with many respondents identifying spending more quality time with their child/family as a positive aspect. As such, we may have inadvertently encouraged parents to engage in more positive appraisal of their overall situation and hence have a lower estimate of their own overall stress. Providing opportunities for cognitive flexibility processes and positive reappraisals is a valuable area for future research, especially while the course of the current pandemic continues and necessitates the return to lockdown in some regions.

## Limitations

It is important to acknowledge the limitations of this research. While a strength of this study is that all participants were resident within a single state and country, and therefore experienced the same implementation of lockdown, the findings may not generalize to residents in other regions where lockdowns have continued for longer, been more strictly enforced, or where a second wave lockdown is experienced. Further, unlike other recent studies examining parenting stress during COVID-19, which have included families with older children (e.g., 0–8 years, Cameron et al., 2020; 2–14 years, Spinelli et al., 2020), our study focused on the experiences of parents who had a child not yet old enough to have started formal schooling in Australia. We acknowledge that the experiences of social isolation will have been more challenging for families experiencing more educational concerns for their school-aged children, as well as in higher risk families such as those with children with additional needs, separated parents, or from more economically disadvantaged areas.

Because of our cross-sectional design, we are also unable to draw conclusions about changes in perceived stress for individuals across the lockdown experience. Although the two waves of data collection (early and late) were from the same region, they were uneven in terms of overall number recruited and source (families with an infant vs. families with any child under 6 years). Other limitations include participant self-selection, and the potential for response biases in online data collection. Finally, our survey respondents were predominately female; therefore, we are unable to draw conclusions about the experiences of fathers. In ongoing research, we are collecting survey and interview data specifically with fathers to better understand their parenting experiences and psychological wellbeing during the pandemic.

## Conclusion

Despite the limitations of online data collection, study findings were able to show that an impact of COVID-19 hardship on parent stress is not universal: instead, the relationship is moderated by the degree to which parents perceive rewards over challenges during lockdown. While lockdowns have disrupted life for millions of people around the globe, and restricted access to social networks and recreational activities that support wellbeing during tough times, the ability to identify positive aspects from this experience appears to play an important role in buffering stress. These findings provide important empirical support for clinical utility in supporting families to develop psychological flexibility during lockdowns. The longer-term impacts of this on family relationships, and the effectiveness for more vulnerable individuals, require further assessment.

## Data Availability Statement

The data supporting the conclusions of this article can be made available by the authors to a qualified researcher upon request.

## Ethics Statement

This study involved human participants and was reviewed and approved by the University of Wollongong Human Research Ethics Committee. The participants provided their written informed consent to participate in this study.

## Author Contributions

JH conceived the study idea. JH, AM, and AB contributed to the conception and design of the study. AM developed the questionnaire. AM and AB coded the data. AB and SB performed the statistical analysis. JH and AB wrote the manuscript. All authors contributed to manuscript revision, read and approved the submitted version.

## Conflict of Interest

The authors declare that the research was conducted in the absence of any commercial or financial relationships that could be construed as a potential conflict of interest.
