# Supplementary material for: Identifying Rewards Over Difficulties Buffers the Impact of Time in COVID-19 Lockdown for Parents in Australia
Source: Front Psychol. 2020 Dec 17;11:606507. doi: 10.3389/fpsyg.2020.606507 (PMC7773812; doi:10.3389/fpsyg.2020.606507)
Supplement: Supplementary file 1 [file Image_1.pdf]

Supplementary Material

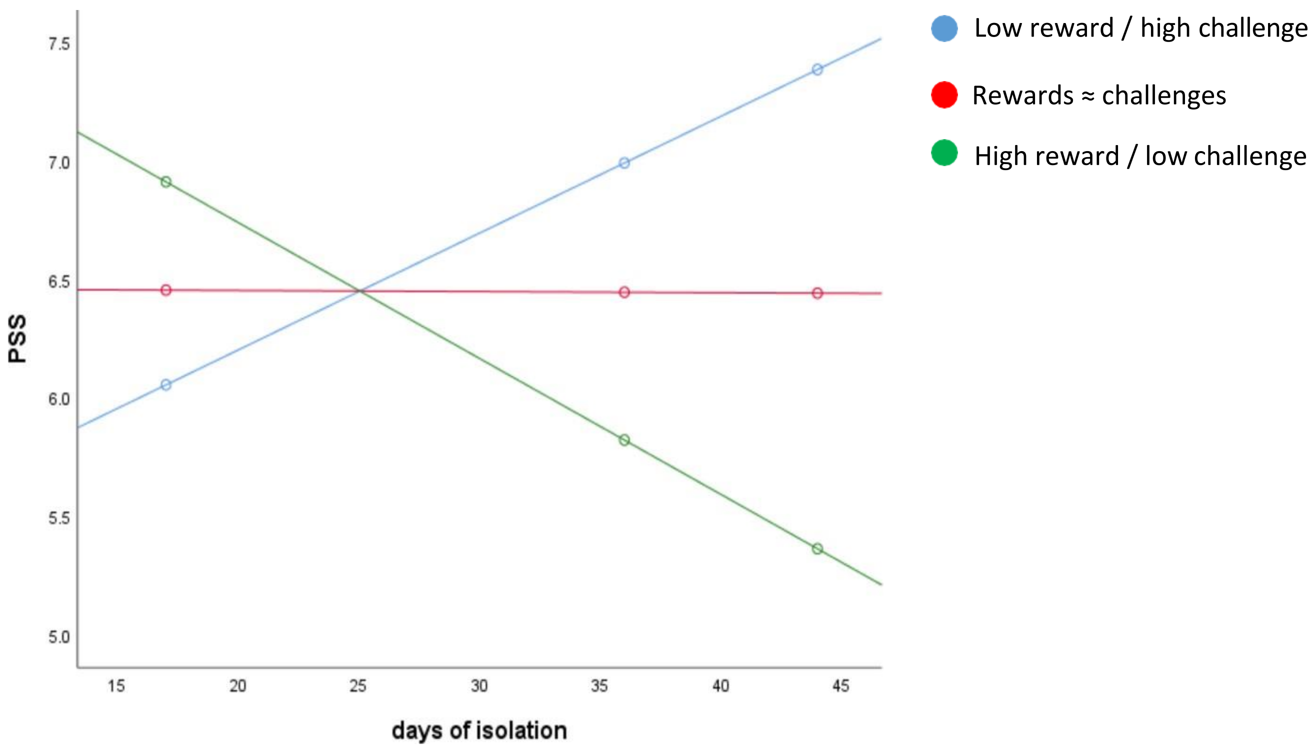

Supplementary Figure 2. Interaction of days in isolation with PSS-4 by parental rewards / challenge ratio
